# Estimated cost and operational structure of pgSIT malaria vector control programs in selected West African countries

**DOI:** 10.1016/j.sciaf.2025.e02888

**Published:** 2025-09

**Authors:** William A.C. Gendron, Robyn Raban, Agastya Mondal, Héctor M. Sánchez C, David Zilberman, Patrick G.C. Ilboudo, Umberto D’Alessandro, John M. Marshall, Omar S. Akbari

**Affiliations:** a School of Biological Sciences, Department of Cell and Developmental Biology, University of California, San Diego, La Jolla, CA 92093, USA; b Divisions of Epidemiology & Biostatistics, School of Public Health, University of California, Berkeley CA, 94720, USA; c Department of Agricultural and Resource Economics, University of California, Berkeley, CA 94720, USA; d African Population and Health Research Center, Nairobi, Kenya; e Medical Research Council Unit The Gambia at the London School of Hygiene and Tropical Medicine, Fajara, Gambia; f Innovative Genomics Institute, University of California, Berkeley, CA 94720, USA

**Keywords:** Malaria, Precision-guided sterile insect technique, Cost-benefit analysis, *Anopheles gambiae*, Value of statistical life, Mathematical modeling, Crispr, Genetic biocontrol, Mass rearing

## Abstract

Malaria control has primarily been achieved through vector control, but current methods are insufficient to achieve elimination. Precision guided sterile insect technique (pgSIT) is a mosquito suppression technique that generates sterile male mosquitoes for mass release. Our previous studies showed that this intervention is expected to be highly cost-effective in a malaria endemic region of West Africa, but these estimates used only 15-31% capacity for sex sorting, which is the limiting production step and a primary cost. We, therefore, determined the most cost efficient facility size by calculating the cost per million *Anopheles gambiae* suppressed as the facility was scaled up to suppress more mosquitoes. We developed an optimized facility size per 9.2 million mosquitoes suppressed, which can be a framework for scaling and increases the cost effectiveness of this intervention. The development of this intervention can potentially interrupt malaria transmission, strengthen local public health institutions, create manufacturing capacity, provide local jobs, and enhance regional health security capabilities that are more resilient to disruptions in supply chains and malaria investment.

## Introduction

Despite substantial efforts over decades, malaria still kills approximately 608,000 people annually [1]. Insecticidal-based interventions targeting the *Anopheles* mosquito vector, such as insecticide treated nets (ITNs)/ long lasting insecticide treated nets (LLINs), and indoor residual spraying (IRS), are currently used to prevent malaria. Major advancements in malaria prevention have been accomplished with these technologies, but further progress requires new approaches. Emerging genetic technologies may help close gaps in the global malaria elimination strategy. These genetic-based tools also provide unique opportunities to improve public health institutions and manufacturing capacity and create jobs in malaria-endemic regions. They also safeguard local health security by developing regional capacity to prevent malaria.

A wide range of genetic technologies kill disease vectors or make them incapable of transmitting disease. The technologies most likely to be approved for wide-scale use in the near future, however, are based on a long-existing technology known as sterile insect technique (SIT), whereby sterile male insects are released frequently in large numbers into the environment to mate with wild females [2]. The offspring of these matings are non-viable, and over many releases, this causes the suppression or elimination of the target population. One clustered regularly interspaced short palindromic repeats (CRISPR)-based SIT technology, precision-guided SIT (pgSIT), has been developed in multiple vector species [[3], [4], [5]] and is a potentially inexpensive means to generate sterile males for release [6]. CRISPR is an RNA-guided DNA-cutting system that can be programmed to create site-specific gene mutations. In a pgSIT context, the CRISPR technology is separated into Cas9 and gRNA lines, which, when crossed, cause disruptions to male fertility and female essential genes, resulting in the production of sterile males. When combined with a fluorescent sex sorting technology, such as SEPARATOR which has been demonstrated in several insects including mosquitoes [[7], [8], [9], [10]], pgSIT can be efficiently and accurately sex sorted at scale. For malaria control, pgSIT is most cost-effective when mosquitoes are manufactured in the endemic region, providing regional infrastructure and economic benefits and improving the resilience, strength, and adaptability of regional public health. During the COVID-19 pandemic, supply chains and malaria prevention programs were disrupted, investment in malaria prevention was reduced, and consequently, malaria cases rose [[11], [12], [13], [14], [15]]. Other global events will likely result in similar outcomes for malaria prevention programs. Therefore, providing regional capability to suppress mosquito populations can provide some resiliency to these programs. Even without these threats, supply chain constraints and access to prevention technologies regularly impede malaria prevention programs. Local production of malaria prevention products, such as mosquito nets [16], for example, can improve accessibility and reduce costs [17].

Regional manufacturing of malaria prevention technologies has added economic benefits, including expanding the public health workforce and regional expertise. pgSIT, in particular, is uniquely positioned to be locally produced as it can be maintained with fairly simple, regionally acquired resources compared to other malaria interventions. We recently conducted a cost analysis of pgSIT to control the malaria vector, *Anopheles gambiae,* in the Upper River Region (URR) of The Gambia [6]. This study showed that pgSIT implemented in the URR is predicted to prevent approximately 230 deaths and over 48,000 sick days per year, saving life years at 15 - 127 USD per life year saved. In addition to the economic benefits of pgSIT, these studies focused on how pgSIT would operate in a malaria-endemic setting, such as the URR of The Gambia, and the investments needed to fund these operations. This previous study does not consider, however, the less measurable pgSIT benefits that make it a promising technology for malaria vector control, the most efficient facility scale to provide the most coverage per dollar, and cost capturing for other countries in West Africa. Therefore, we build upon our previous work to evaluate the optimal production scale and estimate the costs expected for larger scale use in West Africa. We will provide a framework for estimating pgSIT program costs at varying scales, and demonstrate cost savings at larger scales. We will also discuss how the pgSIT program can improve access to malaria prevention technology while reducing cost and ultimately developing a more robust, adaptable, and resilient malaria control program prepared for future disease prevention. We will provide an overview of the cost and structure of a pgSIT program, offer new calculations of expenses and savings when maximizing pgSIT production, and update the pgSIT cost estimates by including cost estimates from other regions in West Africa. Earlier studies discussed only the costs to meet the specific needs of one area, the URR of The Gambia. However, maximizing production can reduce costs and expand the capabilities of pgSIT as a vector control tool. Including a range of expenses expected in West Africa will simplify pgSIT implementation estimates for other malaria-endemic regions and provide the basis for understanding the less measurable benefits of a pgSIT program.

## Methods

### Estimating pre-release field trial costs

**Fig. 1:**
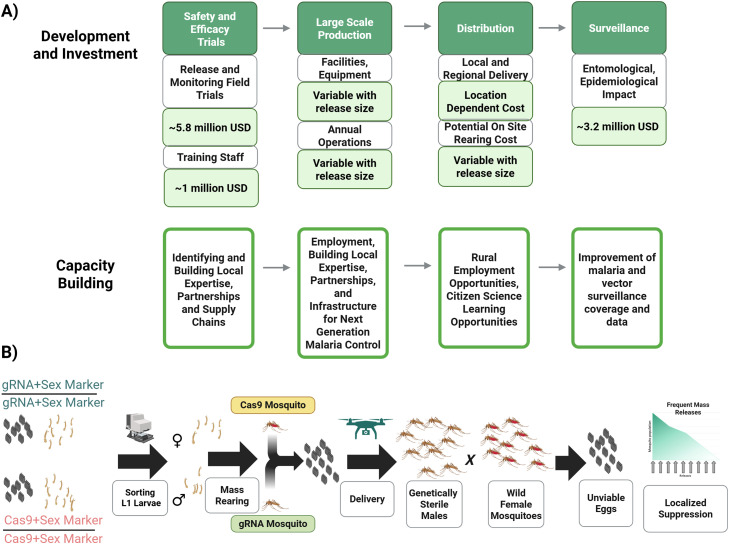
**Development and implementation of pgSIT for malaria control. A)** The flow chart depicts a schematic of the key parts of pgSIT development, investment, and their impacts on capacity building with selected cost estimates for a facility that can suppress up to 9.2 million mosquitoes., These estimates are based on the COPAS sorting device bottleneck and costs. This includes an estimate of initial safety and efficacy trials, and then building the large scale facility and the annual and distribution costs for these releases. Finally, there will be an extended period of surveillance and associated costs to confirm pgSIT's efficacy. Throughout these processes, capacity building will coincide with each of these steps and will be important goals to achieve in order to enable continued development of this intervention. **B)** Schematic of pgSIT production and distribution. Sterile pgSIT eggs are produced by mass-rearing and crossing mosquitoes containing gRNAs targeting genes for female survival and male fertility and CRISPR-associated protein 9 (Cas9) expressing mosquitoes. The female offspring of this pairing are, therefore, killed, and the males are sterilized in the egg stage. These eggs, or any mosquito life stage, can then be distributed to the release sites and maintained to adulthood, where the sterile males can mate with wild female mosquitoes. Female malaria mosquitoes typically mate only once, and if mated with a sterile pgSIT male, they will lay non-viable eggs. These eggs will not hatch, causing a decline in the population as many female mosquitoes fail to produce offspring. Repeated releases of pgSIT eggs can result in localized extinction.

Our previous economic estimates focused on a pgSIT program for the local extinction of *A. gambiae,* the primary African malaria vector. However, like many genetic technologies, pgSIT has yet to be tested outside the laboratory. Preliminary safety and efficacy trials will improve pgSIT performance estimates in the field. This process consists of a series of field experiments of increasing size and scope, with vector population monitoring and, possibly at larger scales, malaria incidence evaluations to determine field efficacy. The initial safety and efficacy trial costs are estimated at 5.8 million USD [6] (Fig. 1A). With scaling of this facility, it is expected that the early trials will not be altered by the location in West Africa nor the size of the facility, but would affect facility function and construction costs. These costs are described in a previous publication and include costs for 2-3 years of cage trials (∼978,000 USD), 1-2 years of small-scale field trials (∼984,000 USD), and the remaining funds for large-scale field trials [6]. These costs will remain unchanged for other locations in West Africa.

### Estimating pgSIT facility startup costs for West Africa

Our previous work focused on evaluating the costs of suppressing *A. gambiae* in one region of The Gambia [6]. We used these estimates and modified them to scale *A. gambiae* production to the full capacity of one Complex Object Parametric Analyzer and Sorter (COPAS) FP 500s (Union Biometrica), which is required for sex sorting the pgSIT Cas9 and gRNA lines, and is the limiting step for scaling pgSIT. With the COPAS being the core technology for these facilities, this estimate includes a spare COPAS device in case a primary COPAS requires repair. These estimates include startup costs, which comprise the expenses for conducting field trials and building and supplying the facility. They were based on local prices when available or adjusted international prices, accounting for currency, importation, and shipping expenses. Estimated costs for field trials and facility start-up were based on prior trials and local research. Estimated costs for a training staff were calculated based on US salaries and Post (Hardship) Differential (DSSR 500) [18]. These estimates were then extrapolated for the suppression of mosquitoes at the full weekly production capacity of one COPAS sex sorter, which is 294,943,361 total eggs. With half of these being inviable females, and a release rate of 16:1 pgSIT:wildtype, this production rate should suppress 9,216,980 mosquitoes. The release rate per wildtype mosquito was previously optimized for cost, as minimizing the weekly volume of mosquitoes released at the expense of extending the number of weeks of releases, which was more cost effective [6]. We utilized a suppression rate of 9,210,000 as this also accommodates scaling adult rearing cages, which are capable of suppressing just over 30,000 mosquitoes per cage. In an effort to capture the range of startup costs for a pgSIT facility in West Africa, we evaluated the malaria endemic countries in this region with highest and lowest GDP per capita, Ivory Coast and Sierra Leone, respectively. In particular, we included differences in the average cost of land per m^2^, additional labor costs for imported training staff based on the Post (Hardship) Differential (DSSR 500) [18], and wages were adjusted per country according to GDP per capita. For example, country-specific differences in salary costs were modified using this formula:

(1) Salaryofacountry=(TheGDPpercapitaofacountry/TheGambiaGDPpercapita)×(TheGambiansalary)

Total cost estimates for each country were calculated for each category and then summed to obtain the total cost. Some of the costs, such as drones, are included as fixed costs between countries. Other costs, such as blood feeding equipment, are considered negligible and were therefore not included. The included production costs were calculated as:

(2) Totalcostforproductionataspecificscale=(Totalnumberofmosquitoestosuppress/Totalcostperunittoproducethatnumberofmosquitoes)×(Costperproductionunit)

To account for the costs of the initial investment, these total costs were also used to derive an annual interest rate of an amortized loan. We utilized a 5% interest rate for these calculations. This was applied over 20 years, as this is a conservative lifespan for SIT facilities. This formula is as follows:

(3) $$\text{Annual}\;\text{interest}\;\text{rate}\;\text{of}\;\text{initial}\;\text{investment}=\left(\text{Initial}\;\text{Cost}\right)\times (0.05{\left(1+0.05\right)}^{20}/\left({\left(1+0.05\right)}^{20}-1\right)$$

Costs were calculated in 2022 USD. Detailed calculations are provided in **Supplemental File 1. Supplemental Table 1** describes the previously established values needed to provide these estimates. **Supplemental Table 2** includes the estimates used to determine the suppression capability per COPAS FP 500 device based on the previously determined release rate. **Supplemental Table 3** and **Supplemental Table 4** estimate the suppression capacity per Wolbaki Mass Rearing Tray and per adult mosquito cage, respectively. These rates are used to calculate the number of COPAS FP 500s, Wolbaki mass rearing trays, and adult mosquito cages included in initial cost estimates of these facilities (**Supplemental Equations 4-7, 18)**. To assess the costs of more efficient facilities, we calculated the costs of a COPAS FP 500 at its full sorting capacity (**Supplemental Table 5)**.

### Estimating annual pgSIT costs for West Africa

Annual expenses to maintain and operate the pgSIT production facility include costs for personnel, supplies, equipment maintenance, and the costs of distributing and conducting surveillance on the program. The increased facility size and production capacity increased production costs and maintenance fees associated with this new scale of facility (Table 2). These quantities were derived from **Supplemental Equations 8-17, 19-20**. These increased costs were applied to the previously established framework and cover maintenance, fuel, salary, water, larval feed, and field rearing costs.

Establishing a functional pgSIT mass-rearing facility requires an initial investment in human capital development. A team of international technical experts will be recruited to design, implement, and troubleshoot operational plans and to train local personnel in pgSIT production. These experts are expected to serve temporarily during the setup and capacity-building phase, and their costs were accounted for in the start-up costs. Post-training facility operations were divided into fixed and variable labor components. The fixed component includes a core management team that is required, regardless of facility scale. The variable labor component scales with operational needs and includes technicians responsible for tasks such as operating equipment and rearing mosquitoes. Each mass-rearing rack requires one dedicated technician, and staffing needs increase linearly with rearing volume. To account for differences in salaries between countries, we applied the ratio of the GDP per capita for Sierra Leone or the Ivory Coast to The Gambia and utilized that ratio to adjust the wages.

Resource requirements for facility operation, including labor, raw materials, and maintenance, were estimated based on prior work [6]. Equipment-specific resource needs were modeled per unit, and maintenance costs were assigned based on either expected fragility (ranging from 1.0% to 10.0% of the initial equipment cost annually) or a manufacturer-provided maintenance fee (12% of the initial equipment cost for the COPAS). An additional initial cost was included to procure spare lasers not covered by the maintenance fee, as was done in our previous study [6]. Onsite release rearing costs were updated to accommodate variable release volumes. These costs include consumables and personnel, determined by the number of active mass-rearing trays. Rearing volume was used as a proxy to calculate labor and material demands. Further optimization and cost modeling will be required if larvae are reared offsite or in alternative rearing environments.

Entomological surveillance costs include trapping and mark-release-recapture (MRR) studies to estimate *Anopheles* population size, dispersal, and survival following pgSIT releases. In large-scale trials, epidemiological monitoring will also assess malaria incidence and infection prevalence to evaluate public health impact. Monitoring parameters will be defined by the trial design and scaled accordingly. Based on prior estimates [6], the combined cost of entomological and epidemiological monitoring during initial trials and five years post-implementation is approximately 3.2 million USD, and will serve as our estimate for other locations in West Africa.

### Estimates of *Anopheles gambiae* suppressed per USD

We estimated the number of *A. gambiae* suppressed per USD by dividing the annual 9.2 million *A. gambiae* suppression capability of a facility by the annual costs or by the annual costs plus the annualized interest rate from the initial costs. This estimate was applied to The Gambia, Sierra Leone and the Ivory Coast.

### Cost per DALY averted

**Fig. 2:**
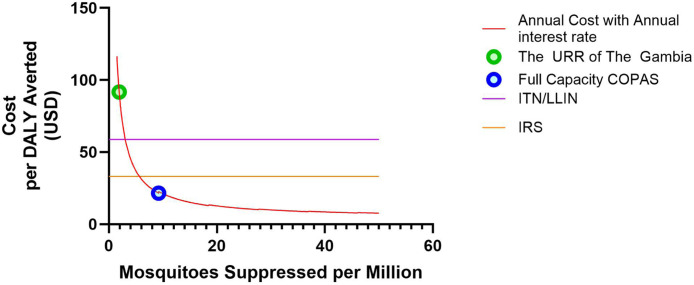
**Cost per DALY averted as production facility is scaled:** The average facility production capabilities and the cost per DALY (Disability Adjusted Life Years) averted were calculated as the facility is scaled. For this calculation, the initial costs were converted to an annual interest of an amortized loan over 20 years. Utilizing this formula, we applied the suppression rates for the Upper River Region (URR) of The Gambia from our previous paper [6] and the optimized facility unit, which utilizes the COPAS sex sorting technology at full capacity. The Costs per DALY averted per million mosquito suppressed points were identified for the URR of the Gambia (green circle) and the optimized facility (blue circle). Conteh et al. [19] was utilized to calculate the average insecticide treated net/long lasting insecticide treated net (ITN/LLIN) and indoor residual spraying (IRS) cost per DALY averted for comparison with the pgSIT costs per DALY averted at a fixed value. These ITN/LLIN and IRS values were derived from studies where these interventions were broadly applied at scale, and these are graphed as fixed values for ease of visual comparison.

We also estimated the cost per Disability Adjusted Life Year (DALY) Averted over a range of mosquito suppression scales, and compared these to average costs per DALY for current interventions, such as ITNs/ LLINs and IRS. To conduct these estimates, we combined the annual costs and annualized interest rate and divided them by the estimated DALY averted. We generated several annual cost estimates and used the mean of these calculations to estimate and graph the ratio of total costs to mosquitoes suppressed (Fig. 2). We utilized the ratio of DALYs averted compared to the total mosquitoes suppressed in our estimate of the URR of the Gambia to derive a rate of 6,191 DALY averted per million mosquitoes suppressed [6]. While we expect this to vary, as there may be regions with different ratios of *A. gambiae* and humans that will alter the cost per DALY, we use this fixed ratio to compare cost efficiency **(Supplemental Equation 21)**. This ratio is likely an underestimate of the average value due to the higher mosquito-to-human ratio in rural areas, such as the URR. The cost per DALY averted was then plotted against the facility's suppression capability to determine the ideal facility size. The previous facility average and the per COPAS Large Particle Flow Cytometer suppression were highlighted. The suppression size per COPAS unit was estimated in **Supplemental Table 1** and the costs were in Table 1. The average annual costs of a facility that utilizes two (one functional and one in reserve) COPAS Large Particle Flow Cytometers to their full capacity are enumerated separately in Table 2. These values were then compared to conventional methods and their ability to save lives in DALY averted in regions with a similar mosquito and human population, and were graphed in Fig. 2. **Supplemental Equation 22** describes the graphed equation and the values input to make Fig. 2 can be found in **Supplemental File 2**. Costs per DALY averted for ITN/LLIN and IRS were utilized from Conteh et al [19] to compare these interventions. All values were converted to 2022 USD.

**Table 1:** Total pgSIT program startup costs for full production capacity, including research and development and initial monitoring (USD).

Location	COPAS	COPAS Spare Lasers	Wolbaki Mass Rearing System	Adult Rearing Cages	Drones	Land and Facility	Initial Trials	Initial Training Staff*	Monitoring	Total Initial Costs
The Gambia	616,348 USD	46,000 USD	202,500 USD	72,250 USD	100,000 USD	688,063 USD	5,801,655 USD	936,000 USD	3,252,500 USD	11,761,316 USD
Sierra Leone						715,427 USD		1,053,000 USD		11,905,680 USD
Ivory Coast						919,279 USD		936,000 USD		11,992,532 USD

This table includes additional costs associated with the larger and more expensive field trials and additional sex sorting equipment needed to scale pgSIT production to suppress 9.2 million mosquitoes. The 9.2 million mosquitoes suppressed is the average expected capability per COPAS sorting device (Union Biometrica) as these devices are a core bottleneck to these production plants and are expensive, which encourages utilizing these devices efficiently. Costs in USD 2022. * Calculated using the State Department’s Post (Hardship) Differential (DSSR 500) [19].

**Table 2:** Annual Costs of 9.2 million suppression capacity facility by country.

Costs/Facility Location	COPAS Maintenance Fees	Rearing Equipment Maintenance Fees	Adult Cage Maintenance Fees	Land and Facility Maintenance Fees	Larval Feed	Water Usage	Drone Delivery	Management, Supervisor Salary*	Non-supervisory Staff*	On Site Rearing Costs	Total Cost	Total Cost with Initial Interest Rate
The Gambia	73,962 USD	2,025 USD	3,613 USD	34,403 USD	543 USD	1,039 USD	10,657 USD	27,824 USD	38,526 USD	94,901 USD	287,493 USD	1,231,251 USD
Sierra Leone				35,771 USD				16,380 USD	22,681 USD	86,308 USD	252,979 USD	1,208,322 USD
Ivory Coast				45,964 USD				85,592 USD	118,514 USD	138,278 USD	480,186 USD	1,442,498 USD

Cost in USD 2022. * Calculated using the State Department’s Post (Hardship) Differential (DSSR 500) [19]. Annual costs and annualized interest rates were applied to these calculations.

Initial Trial Investments include $5,801,655 monitoring: $3,252,500, spare COPAS lasers: $46,000 per laser, 2 required, and Training Staff, which includes Baseline Pay: $78,000 with Hardship Modifiers for The Gambia and Ivory Coast (20%), and Sierra Leone (35%).

## Results

### pgSIT facility start up cost estimates in West Africa

A pgSIT production facility needs to generate sterile male eggs in the quantity required to suppress mosquitoes over the target area (Fig. 1A). These costs include the construction and setup of the facility, necessary equipment, and the investment in experts to support the design and testing of these resources. This cost range is primarily due to the uncertainties associated with scaling mosquito production and sex sorting, but as the early-phase efficacy and safety trials are scaled, these estimates will become more precise. Our previous work estimated the requirements to suppress *A. gambiae* populations in the URR, estimated at about 1.9 million wild mosquitoes per week, a little over one-fifth of the total predicted capability of a pgSIT startup program costing approximately 5.5 million USD [6]. When the rate-limiting parental cross sex-sorting step is at full capacity, pgSIT becomes even more cost-effective with a 9.2 million mosquito suppression capacity corresponding to startup costs of 11.8 million - 12.0 million USD, depending on country (Fig. 1, Table 1). Sex sorting is required to ensure the pgSIT parental lines are reciprocally mated to generate only sterile male offspring (Fig. 1B) and is the limiting step to scaling pgSIT.

### Annual pgSIT facility costs in West Africa

The increased facility size and production capacity resulted in higher production costs and maintenance fees for pgSIT production. For personnel costs, the program will initially require specialized training personnel to develop, implement, and troubleshoot operational plans and train local staff, costing approximately 1 million USD. The facility operations costs include labor, raw resources, and maintenance costs. The operation costs for a pgSIT program to eliminate 9.2 million *A. gambiae* in The Gambia, Sierra Leone, and the Ivory Coast are estimated to annually cost 287,493, 252,979, and 480,186 USD, respectively [6]) (Table 2). When factoring in the initial investment interest rate over 20 years, the costs range from 1,208,322 - 1,442,498 USD. Savings from the economy of scale are expected but difficult to estimate at this stage, so they are excluded from these projections.

### Estimates of *Anopheles gambiae* suppressed per USD

The *A. gambiae* suppressed per dollar was calculated as a standard value for comparison to previous pgSIT cost studies (Table 3). With the facilities at full production capacity per sex sorting device, The Gambia, Sierra Leone, and the Ivory Coast would suppress adult *A. gambiae* at 32.04, 36.41, and 19.18 mosquitoes per annual USD, respectively. When including the interest rate of the initial investment in the annual cost calculation, the suppression rate is 7.48, 7.62, and 6.38 mosquitoes per USD, respectively.

**Table 3:** *Anopheles gambiae* suppressed per dollar, and cost per DALY Averted, at full COPAS capacity facility.

Location	*Anopheles gambiae* Suppressed per dollar (Annual Costs Only)	Cost per DALY Averted (Annual Costs Only)	*Anopheles gambiae* Suppressed per dollar with interest from initial investment	Cost per DALY Averted with interest from initial investment
The Gambia	32.04	5.04 USD	7.48	21.59 USD
Sierra Leone	36.41	4.44 USD	7.62	21.19 USD
Ivory Coast	19.18	8.42 USD	6.38	25.30 USD

Cost in USD 2022. Initial costs included to develop pgSIT for wide scale release.

Wage ratios based on GDP per capita: Sierra Leone to The Gambia: 0.58871, Ivory Coast to The Gambia: 3.0762

DALY averted is an estimate based on the predicted disability-adjusted life year (DALY) averted per mosquito suppressed from Gendron et al. 2025. This ratio was multiplied by the mosquitoes suppressed.

### Cost per DALY averted per million of *Anopheles gambiae*

Comparisons of the DALY averted per million *A. gambiae* suppressed provides an estimate of the cost savings associated with reducing malaria burden, which can be compared to conventional interventions. Using annual costs only, the costs per DALY averted are 2.92 USD and 6.00 USD for Sierra Leone and the Ivory Coast, respectively (Table 3). The costs per DALY averted, including the annualized interest rates for Sierra Leone and the Ivory Coast, are 21.19 USD and 25.30 USD per DALY averted, respectively. The cost per DALY averted for a facility based in The Gambia was graphed per million mosquitoes suppressed to visualize the cost per DALY averted at different production capacities (Fig. 2). We identified the points on this graph that represent the cost per DALY at a suppression level estimated for the URR in our previous work [6] and the optimized pgSIT production based on maximizing the capacity of the COPAS sex sorting system. These comparisons highlight the differences in cost per DALY averted as the level of suppression is scaled. The costs per DALY averted for conventional vector control methods (ITN/LLIN and IRS) were also graphed as horizontal lines for comparison. Detailed calculations and equations are provided in **Supplemental File 1**.

## Discussion

Evaluating pgSIT at more efficient scales has demonstrated that dedicated facilities can compete with existing malaria interventions, offering comparable or lower costs per DALY averted. This scale-up increases the cost efficiency to 2.92 - 25.30 USD per DALY averted compared to the previous study, with a rate of 11 - 94 USD per DALY averted (Table 3) [6]. Notably, facilities that maximize the throughput of the sex-sorting bottleneck significantly reduce costs. Further scaling may enhance efficiency, but optimal deployment will depend on local *A. gambiae* densities, logistical feasibility, political and biological constraints, and financial considerations. Strategic site selection and regional partnerships will be critical to maximizing return on investment. As labor and land costs had minimal impact, pgSIT appears broadly applicable across West Africa, with implementation guided by need and scalability.

Encouraging results from laboratory trials and cost modeling support future field trials to assess pgSIT under real-world conditions [[3], [4], [5],[20], [21], [22]]. These trials will not only evaluate efficacy and safety but also yield critical data on production and scaling performance. Given the high efficiency of fluorescent-based SEPARATOR sex sorting technologies [[7], [8], [9], [10]], combining this technology with pgSIT is a priority for pgSIT programs. While risk remains, SIT-based approaches have a well-established history of being safe and effective. Field trials will also test and reevaluate our production estimates. For example, we can evaluate sex sorting methods under sustained operation to determine even more realistic maintenance needs and throughput limits. As sex sorting is a major cost driver, optimizing this step is crucial for achieving cost-effective scaling. Reducing the cost of the COPAS sex sorting step would make pgSIT even more cost effective for vector control.

Compared to current tools (IRS, ITN/LLINs), pgSIT offers multiple advantages. PgSIT is insecticide-free, species-specific, and not prone to resistance, aligning with the African Center for Disease Control’s One Health goals of minimizing non-target impacts of insecticides and reducing reliance on imported chemicals [23]. Moreover, pgSIT has the potential to reduce vector populations at a lower cost, estimated between 2.92 – 25.30 per DALY averted compared to 33.20 –58.75 for conventional methods. While based on conservative assumptions, these estimates indicate pgSIT’s high cost efficiency, particularly in regions with a lower mosquito-to-human ratio. Additionally, the longer these facilities remain active, the annual interest rate factored in the initial investment will decrease, making this facility even more cost effective per DALY averted. Real-world entomological and epidemiological data will further refine these projections and allow for more accurate DALY averted calculations. Region specific DALY averted estimates can inform optimal facility placement throughout West Africa. The downstream impact on preventive therapies (e.g., SMC, IPTp) may also be significant. If pgSIT drives local vector extinction, it could ultimately replace the need for such interventions in affected regions, yielding further cost savings. pgSIT also has the benefit of providing passive intervention that does not rely on individual behavior to protect communities broadly, both indoors and outdoors. Outdoor biting malaria vectors continue to contribute to malaria transmission; however, conventional IRS and ITN/LLIN technologies are unable to target outdoor biting mosquitoes, resulting in a significant need for technologies to control outdoor biting vectors [24]. Therefore, pgSIT is predicted to save lives beyond what is capable by current interventions, making these underestimates of the impact of pgSIT on human health.

The economic burden of malaria extends beyond direct cases and deaths, hindering regional development and slowing progress on broader public health and humanitarian goals. One estimate attributes a 1.3% reduction in GDP growth in West Africa to malaria, reflecting widespread but hard-to-quantify economic impacts [25]. This burden diverts resources from other critical needs and limits infrastructure development. Eliminating malaria would yield broad, long-term benefits beyond measurable health outcomes. Moreover, investing in local malaria control production capacity offers added regional economic and developmental advantages.

Genetic technologies, such as pgSIT, offer a unique opportunity to build local capacity for malaria control by enabling the production and deployment of vector control tools within endemic regions. Investments in local research, leadership, and workforce development will strengthen malaria prevention programs [17]. Unlike conventional interventions, which depend on centralized production and clinical infrastructure, pgSIT relies on rapid, local distribution of mosquito eggs, favoring in-country facilities. This model supports the growth of regional scientific and technical expertise and provides resilience against supply chain disruptions, as seen during the COVID-19 pandemic. These facilities can serve as hubs for broader vector control efforts, producing sterile males for other regions and vectors (e.g., *A. stephensi, A. aegypti*), and supporting the deployment of future genetic technologies. These facilities will also be built to ACL-2 standards, allowing pgSIT infrastructure to be readily adapted for mass-producing emerging genetic tools, including gene drives or other advanced vector control technologies. Importantly, pgSIT programs foster local capacity in vector control, supporting regional research, workforce development, and scientific self-sufficiency.

## Conclusion

For pgSIT to achieve its full potential, facilities must be 1) self-sustaining and 2) capable of regional expansion. Cost analyses suggest pgSIT could be locally funded, reducing reliance on long-term international support. Like past SIT successes (e.g., New World screwworm), regional suppression of *A. gambiae* may lay the groundwork for continent-wide elimination. The necessary infrastructure will also support future technologies, positioning West Africa at the forefront of innovative, scalable malaria control. While field trials remain, pgSIT’s phased rollout provides a low-risk path toward a transformative vector control solution.

## Author contributions

O.S.A., J.M.M., U.D.A. and D.Z. designed research; W.A.C.G., R.R., A.S., and U.D.A. facility cost assessment; A.M., H.M.S. and J.M.M. mathematical modeling; W.A.C.G., P.G.C.I., U.D.A data collection; U.D.A., O.S.A., and D.Z. edited the paper, W.A.C.G., R.R., and J.M.M. wrote the paper.

## Declaration of competing interest

O.S.A is a founder of Agragene, Inc. and Synvect, Inc. with equity interest. The terms of this arrangement have been reviewed and approved by the University of California, San Diego, in accordance with its conflict of interest policies. All other authors declare no competing interests.

## Appendix Supplementary materials

Image, application 1

Image, application 2
