# Supplementary material for: Estimated cost and operational structure of pgSIT malaria vector control programs in selected West African countries
Source: Sci Afr. 2025 Sep;29:None. doi: 10.1016/j.sciaf.2025.e02888 (PMC12463680; doi:10.1016/j.sciaf.2025.e02888)
Supplement: Supplementary file 1 [file mmc1.docx]

**Supplemental File 1**

**Supplemental Table 1: Values derived from Gendron et al. 2025 [6]**

| Eggs Required per Adult (We require 16 pgSIT males. Half of the eggs are inviable females.) | 32 total eggs |
| --- | --- |
| Daily Copas Sorting Capacity | 562,500 |
| COPAS Weekly Sorting Capacity | 3,937,500 |
| Percentage Rearing Survival | 62.50% |
| Fecundity per Female | 255 |
| Percentage Retained for Colony Maintenance | 6% |
| Total Weekly Egg Production per COPAS | 294,943,359 |
| Suppression Capability per COPAS | 9,216,980 |
| Cost per COPAS | 308,174 USD |
| Total Larvae per Rack | 529,000 |
| Larval Rearing Time (Days) | 8 |
| Weekly Rearing Capacity of 1 Wolbaki Rack | 462,875 |
| Total Weekly Egg Production per Rack | 34,672,230 |
| SuppressionCapability per Wolbaki Rack | 1,083,507 |
| Cost of 1 Wolbaki Rack | 22,500 USD |
| Total Adults per Cage | 16,000 |
| Time in Cage (Days) | 14 |
| Adults per Cage per Week | 8,000 |
| Total Eggs per Week per Cage | 1,020,000 |
| Suppression Capability per Week per Cage | 31,875 |
| Disability Adjusted Life Year (DALY) Averted per Million Mosquitoes Suppressed (Estimate based on the Upper River Region of The Gambia) | 6,191 |
| Annual Management Staff Costs: fixed and comprise the following positions: Set staff of 2 engineers/technicians, 2 logistics managers, 1 factory lead manager, 2 supervisors and 1 drone technician | 27,824 USD |
| Initial Development Costs: 5,801,655, Initial Monitoring Costs: 3,252,500, Initial Training: 936,000, COPAS Spare Laser Cost: 2$\times$ at 46,000 each | 10,082,155 USD |
| Drone Purchase Cost: fixed at the per previous study Gendron et al. 2025 (Assumes 4 drones will provide sufficient coverage for most regions) | 100,000 USD |
| Drone Repair and Fuel Costs: fixed per previous study, Gendron et al. 2025 | 10,657 USD |

Values utilized were derived from Gendron et al. 2025 [6]. Costs in 2022 USD.

**Supplemental Table 2: Mosquito suppression capability with a single-sex sorting device, COPAS FP 500**

| **Daily Sorted Larvae (1)** | **Percentage Rearing Survival (2)** | **Number Surviving to Adulthood Daily (3)** | **Fecundity (Eggs Per Female) (4)** | **Daily Eggs Produced (5)** | **Daily Eggs for Release (6)** | **Weekly Eggs Released (7)** | **Suppression Capability at 32 eggs per adult (8)** |
| --- | --- | --- | --- | --- | --- | --- | --- |
| 562,500 | 50% | 281,250 | 210 | 29,531,250 | 27,759,375 | 194,315,625 | 6,072,363 |
|  |  |  | 300 | 44,296,875 | 41,639,062 | 291,473,437 | 9,108,544 |
| 562,500 | 75% | 421,875 | 210 | 42,187,500 | 39,656,250 | 277,593,750 | 8,674,804 |
|  |  |  | 300 | 63,281,250 | 59,484,375 | 416,390,625 | 13,012,207 |
| **562,500** | **62.50%** | **351,563** | **255** | **44,824,219** | **42,134,766** | **294,943,361** | **9,216,980** |

(1) Sorting rate per hour of COPAS multiplied by 23 hours, with an assumption of 1 hour of maintenance per day.

(2) Rearing survival percentage derived from the International Atomic Energy Agency (IAEA) Mass Rearing of *Anopheles gambiae* Protocol and we apply the average of these as in Gendron et al 2025 [6]. The estimates for daily production capabilities are based on the low (50%) and high (75%) rearing survival estimates, and a range of fecundity estimates.

(3) Survival to adulthood is calculated by multiplying daily sorted larvae by the percentage rearing survival.

(4) Fecundity derived from previous work (Supplemental Table 1).

(5) Daily eggs produced are derived from the number of surviving adults daily divided by two to get the number of adult females, multiplied by fecundity.

(6) Daily eggs for release assume 6% of the eggs daily are needed to maintain the colony at adequate production levels (Supplemental Table 1). Therefore, the daily eggs produced are multiplied by 0.94.

(7) Weekly eggs released are the daily eggs for release multiplied by 7 days/week.

(8) The suppression capability at 32 eggs per adult utilizes the previously determined 16 pgSIT male egg releases per adult mosquito as half of the eggs are inviable females. Therefore, the weekly eggs released are divided by 32 to obtain the suppression capability, which is 32 eggs per adult. These calculations assume the average survival and fecundity of these ranges.

**Supplemental Table 3: Suppression capability per Wolbaki mass rearing tray**

| **Larvae per Rearing System (1)** | **Percentage Rearing Survival (2)** | **Number Surviving to Adulthood Daily (3)** | **Fecundity (Eggs Per Female) (4)** | **Daily Eggs Produced (5)** | **Daily Eggs for Release (6)** | **Weekly Eggs Released (8 days of rearing per system) (7)** | **Suppression Capability at 32 eggs per adult (8)** |
| --- | --- | --- | --- | --- | --- | --- | --- |
| 529,000 | 50% | 264,500 | 210 | 27,772,500 | 26,106,150 | 22,842,881 | 713,840 |
|  |  |  | 300 | 39,675,000 | 37,294,500 | 32,632,688 | 1,019,771 |
| 529,000 | 75% | 396,750 | 210 | 41,658,750 | 39,159,225 | 34,264,322 | 1,070,760 |
|  |  |  | 300 | 59,512,500 | 55,941,750 | 48,949,031 | 1,529,657 |
| **529,000** | **62.5%** | **330,625** | **255** | **42,154,688** | **39,625,406** | **34,672,230** | **1,083,507** |

(1) The total larvae added to a Wolbaki mass rearing system based on the numbers provided by the supplier, Wolbaki.

(2) Rearing survival percentage derived from the International Atomic Energy Agency (IAEA) Mass Rearing of *Anopheles gambiae* Protocol and we apply the average of these as in Gendron et al 2025 [6]. The estimates for daily production capabilities are based on the low (50%) and high (75%) rearing survival estimates, and a range of fecundity estimates. The 62.5% average is applied and utilized throughout this study.

(3) Survival to adulthood is calculated by multiplying daily sorted larvae by the percentage rearing survival.

(4) Fecundity derived from previous work (Supplemental Table 1). Utilizes the 255 average as described in **Supplemental Table 2**.

(5) Daily eggs produced are derived from the number of surviving adults daily divided by two to get the number of adult females, multiplied by fecundity.

(6) Daily eggs for release assumes 6% of the eggs daily are needed to maintain the production as described in Gendron et al, 2025. Therefore, the daily eggs produced are multiplied by 0.94.

(7) Weekly eggs released are the daily eggs for release multiplied by 7 days/week.

(8) The suppression capability at 32 eggs per adult utilizes the previously determined 16 pgSIT male egg releases per adult mosquito, as half of the eggs are inviable females. Therefore, the weekly eggs released are divided by 32 to determine the suppression capability, which is 32 eggs per adult. These calculations assume the average survival and fecundity of these ranges.

**Supplemental Table 4: Estimating suppression capability per adult cage**

| **Adult Mosquitoes per Cage (1)** | **Adults per Cage per Week (14 days in each cage) (2)** | **Fecundity (Eggs Per Female) (3)** | **Weekly Eggs Released (4)** | **Suppression Capability at 32 eggs per adult (5)** |
| --- | --- | --- | --- | --- |
| 16,000 | 8,000 | 210 | 840,000 | 26,250 |
|  |  | 300 | 1,200,000 | 37,500 |
|  |  | 210 | 840,000 | 26,250 |
|  |  | 300 | 1,200,000 | 37,500 |
|  |  | **255** | **1,020,000** | **31,875** |

(1) This is the total adult mosquitoes per mass rearing cage from Gendron et al, 2025.

(2) These cages are maintained for 2 weeks, and therefore we divide the useful capability per week.

(3) Fecundity derived from previous work (Supplemental Table 1). Utilizes the 255 average fecundity (**Supplemental Table 1)**.

(4) Weekly eggs released are derived from the number of adults per cage per week divided by two to get the number of adult females, multiplied by fecundity.

(5) The suppression capability at 32 eggs per adult utilizes the previously determined 16 pgSIT male egg releases per adult mosquito, as half of the eggs are inviable females. Therefore, the “weekly eggs released” are divided by 32 to get the suppression capability at 32 eggs per adult.

**Supplemental Table 5: Initial costs by country at full COPAS FP 500 production capacity**

| **Initial Costs** | **Mosquitoes Suppressed** | **COPAS** | **Wolbaki Rearing System** | **Adult Cages** | **Land and Facility** | **Drone Purchase** | **Initial Trial Inv., Monitoring, Spare COPAS Lasers and Training Staff*** | **Initial Cost** |
| --- | --- | --- | --- | --- | --- | --- | --- | --- |
| Full Capacity the URR of The Gambia | 9,210,000 | 616,348 USD | 202,500 USD | 72,250 USD | 688,063 USD | 100,000 USD | 10,082,155 USD | 11,761,316 USD |
| Full Capacity Sierra Leon |  |  |  |  | 715,427 USD |  | 10,199,155 USD | 11,905,680 USD |
| Full Capacity Ivory Coast |  |  |  |  | 919,279 USD |  | 10,082,155 USD | 11,992,532 USD |

Per unit costs of the equipment are noted in Supplemental Table 1.

*Initial trial investments include monitoring, spare COPAS laser, and staff training costs noted in Supplemental Table 1.

**Supplemental Equations**

Many equations utilize the ceiling notation ⌈x⌉ to round up the nearest integer. This is applied to always round up per device, as once it passes the threshold for that device, an additional device is required. “Mosquitoes suppressed by facility size” is abbreviated to Mosq. Supp. The numbers applied are calculated from previous work and consist of costs or consist of the per device suppression capacity.

COPAS Initial Cost=⌈Mosq. Supp./9216980⌉$\times$ 308174+308174 (**Equation 4**)

Wolbaki Rearing System Initial Cost=⌈Mosq. Supp./1083507⌉$\times$22500 (**Equation 5)**

Adult Cages Initial Cost=⌈Mosq. Supp./31875)⌉$\times$250 (**Equation 6**)

Land and Facility Initial Costs=(⌈Mosq. Supp./31875/10⌉ + ⌈Mosq. Supp./9216980⌉ + ⌈Mosq. Supp./1083507⌉)$\times$10$\times$(1600+8)$\times$1.1 (**Equation 7**)

Annual COPAS Costs=COPAS Costs$\times$0.12 (**Equation 8**)

Annual Wolbaki Rearing System Costs= Wolbaki Rearing System Cost$\times$0.01 (**Equation 9**)

Annual Adult Cage Costs= Adult Cages Cost$\times$0.05 (**Equation 10**)

Annual Land and Facility Costs=Land and Facility Costs$\times$0.05 (**Equation 11**)

Annual Larval Feed Costs=(⌈Mosq. Supp./1083507⌉$\times$1800+5100)$\times$0.0255 (**Equation 12**)

Annual Water Usage=(Mosq. Supp./1083507⌉$\times$9540+18020)$\times$0.01 (**Equation 13**)

Adult Cages Annual Wages=⌈Mosquitoes Suppressed/318750⌉$\times$917 (**Equation 14**)

This equation assumes 1 worker per 10 adult mosquito cages

Wolbaki Rearing System Mgmt Annual Wages=⌈Mosq. Supp./1083507⌉$\times$917 (**Equation 15**)

1 worker per Wolbaki Rearing System

COPAS Annual Wages=⌈Mosq. Supp./9216980⌉$\times$1840+1840 (**Equation 16**)

1 Worker per COPAS

In field Rearing Annual Costs=(Mosq. Supp.$\times$16)/1000$\times$0.01+((Mosq. Supp.16/529000)$\times$1800$\times$0.025)+((Mosq. Supp.$\times$16)/529000$\times$75)+60000 (**Equation 17**)

Initial Costs=Sum of COPAS, Wolbaki Rearing System, Adult Cages, Land and Facility Costs, Drone Purchase and Initial Trial Investments (**Equation 18**)

These costs will vary with suppression capability of the facility.

Annual Cost of Utilizing Facility=Sum of all annual costs (**Equation 19**)

These costs will vary with the suppression capability of the facility.

Annual Cost with Annual Interest Rate=Annual cost+Annual Interest (**Equation 20**)

These costs will vary with the suppression capability of the facility.

Predicted DALY Averted=Millions of Mosq. Supp.$\times$6,191 (**Equation 21**)

6,191 is the DALY Averted per Million Mosq. Supp. rate for the Upper River Region of The Gambia. This is an estimate as this likely will not hold true for other regions, but other regions lack sufficient studies to estimate both the mosquito population and epidemiological factors.

Graphed Equation:

Variable Cost per DALY Averted per Million Mosq. Supp.=(Annual Cost with Annual Interest Rate)/(Predicted DALY Averted per Million Mosq. Supp.$\times$Millions of Mosq. Supp.) (**Equation 22**)

These costs will vary with the suppression capability of the facility.
